# Creating a culture that supports food security and health equity at higher education institutions

**DOI:** 10.1017/S1368980022002294

**Published:** 2023-03

**Authors:** Mateja R Savoie-Roskos, Lanae B Hood, Rebecca L Hagedorn-Hatfield, Matthew J Landry, Megan M Patton-López, Rickelle Richards, Jody L Vogelzang, Zubaida Qamar, Kendra OoNorasak, Georgianna Mann

**Affiliations:** 1 Department of Nutrition, Dietetics and Food Sciences, Utah State University, Logan, UT, USA; 2 Department of Nutrition, Health and Human Performance, Meredith College, Raleigh, NC, USA; 3 Stanford Prevention Research Center, Stanford University, Palo Alto, CA, USA; 4 School of Public Health and Health Science, University of Massachusetts Amherst, Amherst, MA, USA; 5 Department of Nutrition, Dietetics and Food Science, Brigham Young University, Provo, UT, USA; 6 School of Interdisciplinary Health, Allied Health Sciences, Grand Valley State University, Grand Rapids, MI, USA; 7 Family, Interiors, Nutrition and Apparel (FINA), San Francisco State University, San Francisco, CA, USA; 8 Department of Dietetics and Human Nutrition, University of Kentucky, Lexington, Kentucky, USA; 9 Department of Nutrition and Hospitality Management, University of Mississippi, University, MS, USA

**Keywords:** Health equity, Food security, Higher education, College and university students, Food access, Social determinants of health

## Abstract

Food insecurity on college campuses is a major public health problem and has been documented for the last decade. Sufficient food access is a crucial social determinant of health, thus campuses across the country have implemented various programmes, systems and policies to enhance access to food which have included food pantries, campus gardens, farmers’ markets, meal share or voucher programmes, mobile food applications, campus food gleaning, food recovery efforts, meal deliveries and task force/working groups. However, little is understood about how to best address food insecurity and support students who are struggling with basic needs. The impact of food insecurity on students’ academic and social success, in addition to their overall well-being, should be investigated and prioritised at each higher education institution. This is especially true for marginalised students, such as minority or first-generation students, who are at heightened risk for food insecurity. In order to create a culture of health equity, in which most at-risk students are provided resources and opportunities to achieve optimal well-being, higher education institutions must prioritise mitigating food insecurity on the college campus. Higher education institutions could benefit from adopting comprehensive and individualised approaches to promoting food security for marginalised students in order to facilitate equal opportunity for optimal scholastic achievement among students of all socio-demographic backgrounds.

## Position Statement

Sustainable approaches to creating a culture of food security and health equity are essential on college campuses. This type of approach requires moving beyond addressing basic needs^(1,2)^. Instead, the aim would be to create a culture of food security on college campuses that supports students’ nutritional needs, with a focus on health equity which provides ‘the personal agency and fair access to resources and opportunities needed to achieve the best possible physical, emotional and social well-being’^(3)^. Food insecurity manifests in unique ways among students attending institutions of higher education as students rely on negative coping mechanisms which directly impact physical and mental health, academics and social life. As a result, it is important to incorporate individual and comprehensive approaches that involve institutional, state and national policies and systems that influence college students. Furthermore, adopting such transdisciplinary approaches to promoting and improving food security is essential for facilitating equal and optimal scholastic achievement for students at heightened risk of food insecurity. This includes students who identify with one or more of the following characteristics: (1) lower socio-economic status background, (2) has a disability, (3) first-generation student, (4) non-traditionally aged student, (5) international student, (6) non-binary or transgender student and (7) racial or ethnic minority student, (8) has dependents and 9) military-connected students^(1,2,4–7)^. To best improve food security and promote health equity for these students, higher education educators and administrators should use a conceptual framework that employs inclusive and interdisciplinary approaches aligning with the needs of students, which vary from one higher education institution to another.

## Background

Food is a fundamental right for humans and foundational to human well-being. Food insecurity, the inconsistent access to enough food for an active, healthy life^(8)^, is well-established as a barrier to positive health outcomes. This makes food insecurity a crucial social determinant of health to target for public health professionals. Reports of food insecurity on college campuses have been documented for over a decade^(1)^. First thought to be an anomaly, repeated stories of food insecurity among college students have since driven researchers to examine this public health concern on their campuses. In 2019, a multi-institutional survey found that 39 % of the nearly 167 000 college students reported being food insecure in the prior 30 d^(6)^. Similar rates of food insecurity have been found at higher education institutions around the country^(5–7)^. Although rates of food insecurity may be somewhat comparable from one institution to another, food insecurity manifests in unique ways and as a result, a one-size-fits-all approach is not effective at reducing food insecurity among this population^(1,2)^. As a result, campus-specific assessments should be conducted to determine the unique food security needs of each institution.

## Risk factors and impacts of food insecurity

There are many factors that have been associated with increased risk of food insecurity among college students. For example, student classification (i.e. year in school) has been found to be a predictor of food security status, with undergraduate students being three times more likely to be food insecure than graduate students^(9)^. Students who do not participate in a meal plan and those who live off campus may also be at greater risk^(10,11)^.

However, it is important to note that certain populations are more marginalised on campus and in society. Thus, these populations are at heightened risk for food insecurity and should be prioritised to promote health equity in higher education. The following section summarises current research on student categories that experience food insecurity at higher rates than their peers.

International students often experience high rates of food insecurity since they often lack access to resources and employment opportunities as they complete their education abroad^(4)^. First-generation or non-traditional students including military-connected students are more at risk for food insecurity and often lack awareness of available resources or resources are inaccessible due to campus programming that is historically framed around the ‘traditional student’^(12–15)^. Students with dependents or caregiving responsibilities often struggle with food insecurity though single parents of dependent children are especially vulnerable^(16)^. Students with a reported disability may also struggle to access resources due to lack of awareness or hesitation to disclose disability status^(17)^. Minority race heightens the risk of food insecurity among college students and may further intensify the issue if a minority student also identifies with another marginalised group. For example, odds of food insecurity for black, first-generation college students has been shown to be almost 300 % higher than white, first-generation students^(13)^.

Gender non-conforming and transgendered college students face food insecurity rates higher than their gendering conforming and cis-gendered counterparts as these historically marginalised groups may avoid resource seeking due to social stigma^(18,19)^. Students who come from food-insecure households prior to college are also at a higher risk of experiencing food insecurity while attending college^(20)^. In fact, students who experienced childhood food insecurity are over 40 % more likely than their peers to become food insecure^(20,21)^. To complicate matters further, reliance on food aid, credit cards and loans to make ‘ends meet’ while in college can result in the burden of debt persisting even after receiving a college degree, thus continuing the cycle of financial instability^(22,23)^. Interestingly, students receiving financial aid such as Pell Grants are more likely to experience food insecurity both during and after college^(12,22–24)^ with one study linking food insecurity with student loan repayment^(23)^.

The overall impact of food insecurity among college students is still being studied; however, research to date has suggested that food insecurity during college impedes student health and academic success during college. For example, students who report being food insecure are more likely to have diminished academic achievement including lower GPA, behavioural problems, mental health concerns and overall poorer self-rated general health^(1,2,11,15,25)^. Additionally, students report struggling with high levels of stress and disrupted sleep quality which have worsened during the COVID-19 pandemic^(26)^. Social engagement among food-insecure college students may also diminish, increasing the risk for departure from higher education^(27)^. In general, raising awareness on college campuses of this connection between food insecurity and a student’s academic success and overall well-being is an important first step in ensuring food security among students.

## Health equity and food security on college campuses

As higher education institutions continue to promote diversity, equity and inclusion on campus, it is increasingly important to address food insecurity as a means to create health equity among students on campus. Health equity is defined as ‘the personal agency and fair access to resources and opportunities needed to achieve the best possible physical, emotional and social well-being’^(3)^. The unique characteristics of college students, among many other environmental and social factors, should be considered when developing strategies and policies to reduce food insecurity on college campuses. Furthermore, the actual impact of food insecurity on students’ academic and social success, in addition to their overall well-being, should be investigated, monitored and prioritised at each higher education institution. Programmes focussed on retention and successful graduation for high school students, for example, could be used as a model in the college setting in order to foster an atmosphere of health equity for students struggling academically or with basic needs like housing and food^(28)^. Enhancing health equity is especially important because academic performance has been linked to both short- and long-term negative health behaviours^(29)^. Moreover, addressing social determinants of health including access to food, health insurance and healthcare resources like mental health services are fundamental in reducing health disparities among college students^(30)^.

Providing educational opportunities such as service learning to engage students with issues related to social determinants of health and health equity could be beneficial^(31)^, as college students are knowledgeable of those terms but have negative attitudes and beliefs, calling them ‘political’ and ‘divisive’ terms^(32)^. To take it one step further, higher education institutions could benefit from adopting comprehensive, student-centered approaches to promoting food security and health equity for historically marginalised students in order to facilitate the equal opportunity for optimal scholastic achievement among students of all socio-demographic and racial/ethnic backgrounds.

## Utilising a framework for improving campus culture

Because food insecurity among students at higher education institutions is multifaceted and complex^(1,2)^, it is beneficial to the campus community to utilise a comprehensive equity framework to ensure all aspects of food security are being addressed. The Health Equity Framework (HEF), a justice-based scientific model, has been developed to conceptualise the complex interaction between people and their environment which impacts health outcomes^(3)^. Unlike other frameworks which target personal barriers to achieving health equity, the HEF focuses on the population level to target factors that impact availability and access to programming and individual agency to utilise resources. Details on the HEF have been published by the creators of the framework elsewhere in more detail^(3)^ but in short encompasses four interacting spheres of influence: (1) relationships and networks, (2) systems of power, (3) individual factors and (4) physiological pathways. Below we highlight areas for improvement in each sphere of the framework which can be utilised to create a culture that supports food security and health equity on college campuses.

## Relationships and networks

Campuses across the country have already implemented a wide range of programming to aid students in accessing food to alleviate food insecurity, such as food pantries, campus gardens, farmers’ markets, meal share or voucher programmes and mobile food applications^(1,2,33)^. There are also student-led food insecurity initiatives and programmes, including campus gleaning, food recovery efforts, food lockers and meal deliveries^(1,33–35)^. Although these campus-based initiatives exist and are growing in popularity, little data are available on the impact these programmes have on the food security status of participants^(1,2,36,37)^ since evaluation strategies across campuses are inconsistent^(38)^. Furthermore, college students have voiced the need for further interventions to address inadequate financial resources, unrealistic food costs on campus, meal plan inflexibilities and the need to learn life skills while in college^(36)^. Some higher education institutions have established working groups or task forces to coordinate and oversee initiatives around meeting the basic needs of their students^(38,39)^. In some circumstances, colleges and universities have hired dedicated staff member(s) or assigned existing staff to lead campus initiatives or serve as points of contact to help food-insecure students find assistance^(33,38,39)^.

Further, improving the overall food environment including type, amount and availability of food on campus is also essential for creating an environment that supports food security^(1)^. The ‘optimal campus food environment’ will depend on the unique aspects of the higher education institution. For example, urban campuses may rely on commercial food establishments to augment food security through food donations or acceptance of food vouchers, while rural campuses may depend more on community or religious organisations, area farmers or focus on more intra-institution programmes.

Overall, the tenacity of this problem requires a more nuanced, integrated and collaborative approach that leverages a wide range of resources to mitigate food insecurity and better equip students for academic success and holistic health. Developing relationships with key stakeholders, which likely differ between institutions, is a needed step to promoting an environment that supports marginalised students and connects them to valuable resources. For example, it would be advantageous for collaboration to occur across disciplines (i.e. nutrition, dietetics, social work, public health/health promotion, psychology, academic services, etc.) and institutional departments (i.e. student services, financial aid office, counseling center, affinity groups and on-campus health centers) to ensure a comprehensive approach is used that targets each area of health, wellness and academic performance that can be influenced by food insecurity^(40,41)^.

## Systems of power

While collaborative grassroots efforts are important, it is ultimately university leadership who is responsible for driving the campus culture and ensuring these programmes flourish. Administration and higher education stakeholders hold the power to allocate resources and opportunities directly to groups identified at higher risk of food insecurity. Higher education institutions should consider the economic components (e.g. income, purchasing power and affordability), the physical components from the perspective of the built environment and physical abilities (e.g. transportation and access) and the socio-cultural components (e.g. stigma, embarrassment and labeling) that are associated with food insecurity^(42)^.

Local, state and national policies play a significant role in food security of college students^(1,2,43)^. Higher education institutions have the opportunity to raise awareness of how these policies influence food-related resources that may be available to students^(44)^. For example, the majority of able-bodied college students are not eligible for government food-purchasing assistance programmes like the Supplemental Nutrition Assistance Program (SNAP); however, there are circumstances when students are eligible for SNAP^(43,45)^. Yet, less than half of eligible college students actually participated in the SNAP programme in 2016^(43)^. More students were able to gain temporary eligibility during the COVID-19 pandemic though this temporary relief is expected to terminate at the end of the Public Health Emergency^(45)^. In addition, higher education institutions that have greater numbers of non-traditional students are more likely to have a greater percentage of students who are eligible for programmes such as SNAP or the Special Supplemental Nutrition Program for Women Infants and Children (WIC). Although these programmes are not implemented on college campuses, it would be advantageous for higher education institutions to raise awareness of circumstances when federal nutrition assistance programmes may be resources for eligible students and to provide support for the application processes^(1,2,43–45)^.

## Individual factors

Surprisingly, a general lack of awareness exists among students regarding available food resources on campus^(1,2,43,44)^. Thus, more should be done to raise awareness of federal nutrition assistance programmes and improve food insecurity policies and programmes on campuses. In the circumstance that awareness exists, stigma is also a known barrier to accessing resources^(1,2,4,43)^. Some campuses have intentionally aimed to mitigate stigma by centralising their food pantries in visible locations on campus^(35,46)^; however, more needs to be done to ensure these campus programmes are socially acceptable by marginalised students. In general, efforts should include education to students and other campus stakeholders on the impact of unmet basic needs, such as access to safe, affordable and healthy food, on academic success and health outcomes^(4)^. Apart from the direct outreach to students, more comprehensive awareness efforts should include a robust social media presence, updated online content and campus-wide campaigns^(44)^. A combination of outreach, education and dissemination efforts needs to be implemented to raise awareness about the issue of food insecurity among students and to promote available resources and programming.

## Physiological pathways

Higher education administration, faculty and staff would benefit from increased awareness of the toll food insecurity takes on a college student’s academic, social and physical/mental health outcomes^(1,2)^. Experiencing food insecurity throughout childhood is related to lower cognitive performance^(47)^ with similar indications shown when food insecurity is present in higher education^(1,2)^. As identified by the HEF, adverse childhood experiences disrupt many cognitive functions which likely transcend over time^(3)^. These early experiences may result in marginalised student populations having different needs than traditional students. Academically, food-insecure students may struggle as a result of poor nutritional quality or overall intake^(48)^. Further, the cognitive burden of the stress faced by food-insecure students may make academic endeavours a challenge^(15,27)^. Diverse and flexible pedagogies that meet marginalised students’ needs are vital but require effort and buy-in from faculty and administration.

Food insecurity during college can also result in students becoming isolated from social networks due to embarrassment or inability to afford social events^(1)^. Lack of social engagement during college heightens the risk for departure from higher education with marginalised students feeling the biggest impact of leaving higher education without a degree. Thus, providing social networks for marginalised students should be a priority.

Lastly, historic and current experiences of food insecurity can result in poor mental and physical health which can contribute to further decline in academic and social engagement^(1,2,15)^. As the psychological components of food insecurity are multifaceted and interrelated, a holistic approach may be of value on campus as a ‘one stop shop’ for support for marginalised students. Holistic programming and interventions to support marginalised students’ specific needs are warranted.

## Recommendations for higher education institutions

We propose that higher education institutions use a multidimensional approach addressing each of the spheres of the HEF to improve food security and health equity on their campuses by:

- Raising awareness among students, educators, administrators and policymakers of the extent to which food insecurity exists and manifests at higher education institutions. (HEF Spheres: All)
- Assessing the factors that contribute to food insecurity among the student population at each institution to guide a strategic plan of policy and programme solutions. (HEF Spheres: Systems of Power)
- Increasing awareness of existing resources on campus as well as community-based resources like SNAP and WIC while promoting the process for applying and utilising these resources. (HEF Spheres: Relationships and Networks, Individual Factors)
- Developing comprehensive and interdisciplinary approaches to promote and improve food security and health equity at higher education institutions based on campus data regarding the need and contributing factors. (HEF Spheres: Systems of Power, Individual Factors, Physiological Pathways)
- Supporting and initiating institutional, state and national policies that aim to improve food security and health equity among students at higher education institutions. (HEF Spheres: Systems of Power)
- Expanding and diversifying the research on food insecurity at higher education institutions to include the impact and use of specific policies, systems and programmes aimed at minimising campus food insecurity. (HEF Spheres: Systems of Power, Relationships and Networks)
- Addressing social determinants of health and enhancing health equity through programmes and policies aimed at students most at risk for food insecurity and health disparities. (HEF Spheres: Systems of Power, Individual Factors)
- Providing adequate campus resources to support the holistic health of college students including expanded access to mental health resources. (HEF Spheres: Systems of Power, Physiological Pathways)
- Creating educational opportunities to address negative perceptions and stigmas around basic need insecurities and health equity issues. (HEF Spheres: Individual Factors)
- Integrating process and outcome evaluation methodologies to identify programme features that need modified or realigned to equitably meet food security and nutritional needs of college students. (HEF Spheres: Systems of Power, Individual Factors)
- Partnering with nutrition, public health and other departments on campus to involve students and faculty in food security initiatives and possibly the inclusion of food/health equity projects in the curriculum. (HEF Spheres: Relationships and Networks)

## Summary

Risk factors for college food insecurity include classification as a first-generation, nontraditional, international student or military connected-student; disability status; having dependents; identifying as gender non-conforming or transgendered or part of a racial, ethnic or minority group^(1,2,4–7)^. Campuses across the country have implemented a wide range of programming to improve food security such as food pantries, campus gardens, farmers’ markets, meal share or voucher programmes, mobile food applications, campus food gleaning, food recovery efforts and meal deliveries while some higher education institutions have relied on working groups or task forces to further these efforts^(1,33–38)^.

More can be done to raise awareness of federal nutrition assistance programmes and improve food insecurity policies and programmes on campuses. The impact of food insecurity on students’ academic and social success, in addition to their overall well-being, should be investigated and prioritised at each higher education institution to create a culture that supports marginalised students and promotes health equity. Higher education institutions could benefit from adopting comprehensive and individualised approaches that address the four spheres of the HEF for their unique campus and student populations. By expanding resources and supports to create a more nutritionally secure environment, historically marginalised students gain equal opportunity for optimal scholastic achievement among students of all socio-demographic backgrounds. Higher education instructors and administrators should consider using a conceptual framework, such as the HEF, to employ inclusive and interdisciplinary approaches aligning with the needs of food-insecure students, which vary from one higher education institution to another.
